# Correction: Coxon et al. Preliminary Data about Habitat Use of Subadult and Adult White Sharks (*Carcharodon carcharias*) in Eastern Australian Waters. *Biology* 2022, *11*, 1443

**DOI:** 10.3390/biology11121762

**Published:** 2022-12-05

**Authors:** Jessica L. Coxon, Paul A. Butcher, Julia L. Y. Spaet, Justin R. Rizzari

**Affiliations:** 1 School of Life and Environmental Sciences, Deakin University, Waurn Ponds, VIC 3216, Australia; 2 New South Wales Department of Primary Industries, Fisheries, National Marine Science Centre, Southern Cross University, Coffs Harbour, NSW 2450, Australia; 3 Evolutionary Ecology Group, Department of Zoology, University of Cambridge, Cambridge CB2 3EJ, UK

## Error in Figures

In the original publication [1], there was a mistake regarding Figure 1 and Figure 2 as published. The journal published a low-resolution copy of both figures instead of the supplied high-resolution copy. The corrected Figure 1 and Figure 2 appear below.

## Text Correction

There was an error in the original publication. The term ‘New South Wales’ was used. A correction has been made to *Results*, *3.2. Horizontal Movement and Behaviour*, *paragraph 2*:

In the sentence starting with “W2 entered shelf waters ~3 km from North Stradbroke Island, New South Wales, ~160 km north……” on line 7, replace the words “New South Wales” with “Queensland”. It will then read “W2 entered shelf waters ~3 km from North Stradbroke Island, Queensland, ~160 km north……”.

The authors state that the scientific conclusions are unaffected. This correction was approved by the Academic Editor. The original publication has also been updated.

## Figures and Tables

**Figure 1:**
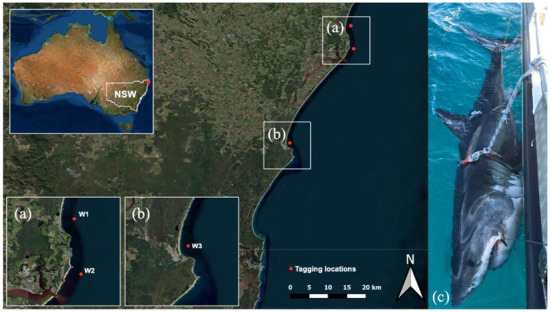
Location of tagging sites (red dots) on the northern New South Wales coast of eastern Australia. (**a**) Lennox Head, tagging site of white shark 1 (W1) and Ballina, tagging site of white shark 2 (W2), (**b**) Evans Heads, tagging site of white shark 3 (W3), and (**c**) W1 secured to the side of the vessel during tagging procedure (image provided by the New South Wales Department of Primary Industries). Map generated in QGIS.

**Figure 2:**
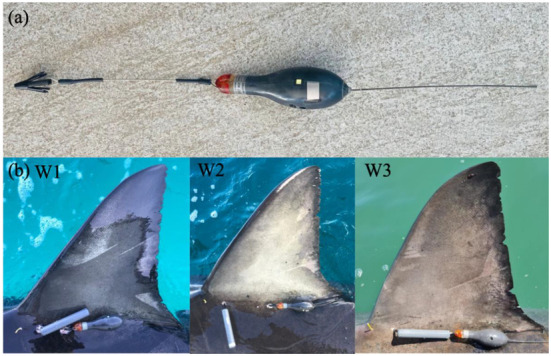
(**a**) MiniPAT-348 Pop-up Satellite Archival Transmitting Tag and Domeier dart head used to anchor the MiniPAT tags and acoustic transmitters into the sharks musculature, and (**b**) dorsal fins of the three white sharks used in the present study. Images taken after completing tagging procedure, with MiniPAT tags, acoustic transmitters and conventional tags visible (images provided by the New South Wales Department of Primary Industries).
